# Psychometric evaluation of an electronic Asthma Symptom Diary for young children

**DOI:** 10.1186/s41687-023-00647-y

**Published:** 2023-10-30

**Authors:** Valerie Williams, Carla Romano, Marci Clark, Dane Korver, Nikki Williams, Diana Goss, Christel Naujoks, Jessica Marvel

**Affiliations:** 1 RTI Health Solutions, Research Triangle Park, NC USA; 2 RTI Health Solutions, Ann Arbor, MI USA; 3 Novartis Pharma AG, Basel, Switzerland; 4 Novartis Pharmaceuticals, East Hanover, NJ USA; 5 RTI Health Solutions, Research Triangle Park, NC USA

**Keywords:** Electronic pediatric Asthma symptom diary, Psychometric evaluation, Patient-reported outcomes

## Abstract

**Background:**

Patient-reported outcome measures that facilitate self-report by children are needed to reduce the bias of proxy report. We previously developed an electronic Pediatric Asthma Symptom Diary (ePASD) to assess the severity of daily asthma symptoms and proximal impacts in children aged 6–11 years with mild to severe asthma. The ePASD, administered via a digital application with visuals, sounds, and text, is uniquely designed to minimize the importance of reading skills on children’s ability to self-report accurately. Here, we describe the ePASD’s psychometric properties.

**Methods:**

Ninety-one children aged 6–11 years with mild to severe asthma and their caregivers participated in 2 study visits, which consisted of training on the provisioned device and completing asthma-specific clinical outcome assessment (COA) questionnaires. The children self-completed the ePASD at home twice daily for 8 consecutive days. The scoring of the ePASD was guided by factor analyses, inter-item correlations, and internal consistencies. Reliability, discriminating ability, construct validity, and responsiveness were evaluated for ePASD items and candidate scores.

**Results:**

All COAs included in the study—the ePASD, Asthma Control Questionnaire (ACQ), Childhood Asthma Control Test, Pediatric Asthma Quality of Life Questionnaire–Standardized (PAQLQ[S]), and global ratings—demonstrated that the children exhibited few asthma-related symptoms and impacts at all timepoints, and consequently, showed little change over time. Internal consistencies (all Cronbach’s alphas ≥ 0.52) and test-retest reliabilities (all intraclass correlation coefficients ≥ 0.60) were largely satisfactory. Patterns of convergent and divergent correlations supported the construct validity of ePASD scores. The ePASD symptom scores correlated moderately to strongly with PAQLQ(S) Symptom scores (all correlations ≥ − 0.46) and with ACQ scores (all correlations ≥ 0.42), as predicted. Evidence of the discriminating ability of ePASD items and composite scores was demonstrated by known-groups analyses.

**Conclusions:**

The ePASD is a reliable and valid measure of asthma symptoms and proximal impacts in children aged 6–11 years with mild, moderate, or severe asthma. These results lay the psychometric groundwork for use of the ePASD in future clinical trials for the management of pediatric asthma. An ongoing pediatric asthma treatment trial is anticipated to provide evidence of the ePASD’s responsiveness to change.

**Supplementary Information:**

The online version contains supplementary material available at 10.1186/s41687-023-00647-y.

## Background

Childhood asthma is a chronic respiratory disease characterized by symptoms of cough, wheeze, chest tightness, and difficulty breathing, which have a considerable impact on a child’s daily activities and quality of life [1]. Asthma is the most common chronic disease among children [2], with an estimated prevalence of 8.1% in children aged < 18 years in the United States (US) [3]. Increases in the global prevalence of childhood asthma, along with related increases in morbidity and mortality, drive the need to develop effective treatments to reduce this burden [4].

As part of treatment evaluation in clinical trials, asthma symptoms are generally assessed using a daily diary; however, these data are typically observer reported for younger children. The observed discordance between self-report from children and proxy report from caregivers highlights the need for a single measure that allows self-report for younger children [5–7]. To meet this need, we developed the electronic Pediatric Asthma Symptom Diary (ePASD) to assess the severity of daily asthma symptoms and proximal impacts in children aged 6–11 years with mild to severe asthma [8]. The ePASD was developed in accordance with US Food and Drug Administration (FDA) guidance and ISPOR good research practices [9–11]. The unique design of this electronic patient-reported outcome (PRO) measure allows it to be administered via a digital application incorporating age-appropriate audio and visuals, including written text with voiceover, thereby minimizing the importance of reading skills, facilitating children’s ability to self-report accurately, and reducing the bias of proxy report.

The anticipated context of use of the ePASD is in children with asthma aged 6–11 years participating in clinical trials evaluating new treatments, enrolled in observational studies, or being seen in clinical practice. To support the use of the ePASD for this purpose, a comprehensive psychometric evaluation in alignment with PRO guidance [9] is required. Accordingly, the objective of this study was to perform an initial psychometric evaluation of the ePASD in children aged 6–11 years with mild to severe asthma. Here, we describe the results of the evaluation of the ePASD’s structure, scoring, reliability, validity, and responsiveness.

## Methods

### Study design

This psychometric evaluation study utilized a prospective, observational, longitudinal design that received ethics approval from the RTI International Institutional Review Board (Federal-Wide Assurance #3331). Eligible pediatric participants aged 6–11 years with mild to severe asthma and their primary caregivers were recruited through qualitative research facilities in the US. All potential participants were screened for eligibility according to the criteria presented in Table 1. Screening was performed via telephone by trained staff at the qualitative research facility using a recruitment screener, which was reviewed with the caregiver of the child with asthma.

**Table 1:** Eligibility criteria

Inclusion Criteria, Pre and Post Study Pause
▪ Signed consent, assent, and parent permission
▪ Aged between 6 and 11 years, inclusive
Have mild to severe persistent asthma diagnosed by a physician ≥ 6 months ago as defined by Global Initiative for Asthma (GINA) (2019) guidelines: • Mild asthma: asthma that is well controlled with Step 1 or Step 2 treatment • Moderate asthma: asthma that is well controlled with Step 3 • Severe asthma: asthma that requires Step 4 or Step 5
Have experienced at least 1 or more of the following in the past 4 weeks: • Daytime asthma symptoms more than twice per week • Nighttime waking because of asthma • Use of reliever (rescue medication) for asthma symptoms (excluding use of reliever before exercise) more than twice per week • Any activity limitation because of asthma
▪ Taking at least a low dose of inhaled corticosteroid (symptom driven in mild asthma, consistent with GINA [2019] Step 2 treatment) for ≥ 3 months
▪ Able to understand and provide responses in English
▪ Have an adult primary caregiver (provides daily care) who is able to read, understand, and provide responses in English and is willing to participate in the study with their child
**Additional Post-Pause Inclusion Criteria**
▪ Access to a computer/laptop to complete digital PDF questionnaires and return by email to the research facility
▪ WiFi access to support daily child ePASD completion using the provisioned device
**Exclusion Criteria**
▪ Patients with a history of chronic pulmonary disease other than asthma, or another condition that affects lung function
▪ Participants who, in the opinion of the qualitative research facility staff or caregiver, were unable to comply with the study procedures or who have any medical or mental disorder, situation, sensory deficit, or diagnosis that could interfere with the proper completion of the study

ePASD = electronic Pediatric Asthma Symptom Diary

The target sample size of 100 was based on the projected acceptable precision (90% confidence interval half-width of 0.10) around an expected test-retest reliability intraclass correlation coefficient (ICC) ≥ 0.70, which is generally taken to indicate adequate test-retest reliability [12]. Recruitment targets were as follows: (1) at least 25 participants from each disease severity level (mild, moderate, severe) across the total sample; (2) at least 10 participants representing each age (6, 7, 8, 9, 10, and 11 years) across the total sample; and (3) at least 15 participants who cannot read English independently. To potentially enrich our ability to observe change, we also sought to recruit both participants who were “Stable,” defined as participants with no changes in asthma medications in the last 2 weeks, and participants who were “Not stable,” defined as participants who required a medication change to improve asthma symptoms in the last 2 weeks.

The study commenced on 3 March 2020 but was paused on 14 March 2020 due to the coronavirus disease 2019 (COVID-19) global pandemic and its health risks for children with asthma. Prior to the study pause, child and caregiver participants attended 2 study visits in person at the qualitative research facility. The study resumed on 7 April 2021 with virtual data collection procedures only (i.e., no in-person study visits), including 2 virtual study visits. Both the in-person (pre-pause) and virtual study visits at baseline (Day 1) entailed research facility staff (1) obtaining each participant’s written informed consent (paper or electronic PDF); (2) training participants on the provisioned device, which was used for the child’s completion of the ePASD; and (3) administering asthma-specific clinical outcome assessment (COA) questionnaires. To facilitate virtual data collection after the study pause, participants were emailed PDF versions of the COA questionnaires and mailed the electronic device (a tablet) prior to the Day 1 virtual study visit, which were conducted via web-based (i.e., Zoom) meetings.

Table 2 describes the schedule of data collection, including at-home ePASD completion. Beginning with the evening of Day 1, child participants were required to complete the ePASD at home twice daily for 8 consecutive days using the provisioned tablet. End of study (EOS) was defined as the day of the second study visit, when participants completed the final set of COA questionnaires and returned their electronic devices, which occurred on the day of the final completion of the nighttime ePASD items. Data were collected from all participants for up to 9 days (± 2 days) because at least 7 days of data for each subject at baseline and EOS were desired for analysis and because it was feasible to schedule study visits on weekdays only (Monday through Friday). Prior to the study pause, child and caregiver participants completed a final set of COA questionnaires and returned the tablet in person on Day 9 (± 2 days). Following the study pause, participants completed a PDF version of the final set of COA questionnaires on Day 9 (± 2 days) and returned the tablet via US mail. All child participants continued to receive their normal medical care and asthma treatment, which were not influenced by the observational study protocol.

**Table 2:** Schedule of key events for ePASD validation analyses

Procedures	Screening	Study visit^a^ 1 Day 1	Study Day 1 to EOS Day −1	EOS visit^a^
Screening, demographics (participant and caregiver), and medical history (participant)	X			
Informed consent and pediatric assent		X		
ePASD			PM and AM	
PAQLQ(S), ACQ-5, ACQ-IA-5, ACQ-IA-6, C-ACT, PGIS, and CGIS		X		X
PGIC and CGIC				X

ACQ-5 = Asthma Control Questionnaire, Symptoms Only; ACQ-IA-5 = Asthma Control Questionnaire–Interviewer Administered, Symptoms Only; AM = completed in the morning; C-ACT = Childhood Asthma Control Test; CGIC = Caregiver Global Impression of Change; CGIS = Caregiver Global Impression of Severity; EOS = end of study; ePASD = electronic Pediatric Asthma Symptom Diary; PAQLQ(S) = Pediatric Asthma Quality of Life Questionnaire–Standardized; PGIC = Patient Global Impression of Change; PGIS = Patient Global Impression of Severity; PM = completed in the evening

^a^ In-person study visit for pre-pause sample; virtual study visit for post-pause sample

### Outcome measures

The psychometric analyses focused on the ePASD, an electronic diary designed to facilitate the daily self-report of asthma symptoms, proximal impacts, and rescue medication use by children with asthma aged 6–11 years [8]. The ePASD includes a nighttime diary that is completed each morning and a daytime diary completed each evening. The nighttime diary comprises 5 items assessing nocturnal asthma symptoms (cough, wheeze, difficulty breathing), nighttime awakening due to asthma, and rescue medication use; the daytime diary comprises 7 items assessing daytime asthma symptoms (cough, wheeze, chest pain/tightness, difficulty breathing), activity limitations, and rescue medication use. The ePASD employs a variety of response formats, including yes/no questions, 4-point ordered rating scales, and 0-to-8 response scales for the number of daytime and nighttime rescue inhaler puffs. For analysis purposes, the ePASD symptom items were scored such that the absence of the symptom (e.g., “I didn’t cough,” “I didn’t wheeze”) = 0, “A little bad” or “A little hard” = 1, “Bad” or “Hard” = 2, and “Very bad” or “Very hard” = 3. The 2 ePASD items assessing the frequency of rescue medication use (i.e., “How many puffs of your rescue inhaler did you take today?” and “How many puffs of your rescue inhaler did you take last night?”) were used to define a rescue medication–free day (RFD), a dichotomous variable indicating whether a participant used a rescue medication that day (flag = 0) or not (flag = 1). The ePASD items are presented on an electronic platform (i.e., a tablet application) using age-appropriate interactive multimedia, including cartoon visuals and audio capabilities. This enables young children with limited or no reading skills to self-report, thereby facilitating the capture of the pediatric patient perspective and reducing bias from caregivers. Within the ePASD electronic data collection tool, children could only select the available buttons associated with specific response options (e.g., “I didn’t cough,” “A little bad,” “Bad," or “Very bad”), eliminating out-of-range item-level responses and outliers; the Next button was disabled within the ePASD until a response button was clicked, eliminating item-level missing data within each completed questionnaire. Skip patterns were programmed into the ePASD so that children were not asked irrelevant questions. 

The performance of the ePASD was evaluated using additional COA measures designed to assess asthma control and asthma symptom severity. The following additional measures were included in this study: Pediatric Asthma Quality of Life Questionnaire – Standardized (PAQLQ[S]) [13]; Asthma Control Questionnaires (ACQ), including ACQ Symptoms Only (ACQ-5) [14] for the pre-pause sample, ACQ Interviewer Administered, Symptoms Only (ACQ-IA-5) [15] for the pre-pause sample, and ACQ Interviewer Administered 6 (ACQ-IA-6) for the post-pause sample; Childhood Asthma Control Test (C-ACT) [16]; Patient Global Impression of Severity (PGIS); Patient Global Impression of Change (PGIC); Caregiver Global Impression of Severity (CGIS); and Caregiver Global Impression of Change (CGIC). The PAQLQ(S) was developed to measure health-related quality of life in children with asthma. The ACQ-5 was designed to measure the adequacy of asthma control and change in asthma control in older children and adults, while the ACQ-IA-5 and ACQ-IA-6 were developed for children aged 6 to 10 years. The C-ACT was designed to assess asthma control in children aged 4 to 11 years. The PGIS assessed participants’ current asthma symptom severity using a 4-point verbal rating scale for the question “How bad are your asthma symptoms right now?” (0 = “I don’t have asthma symptoms right now,” 1 = “A little bad,” 2 = “Bad,” 3 = “Very bad”). The PGIS was completed by all child participants during the study visits on Day 1 and EOS. The PGIC assessed change in asthma symptom severity from the patient perspective using a 5-point graded response scale for the question “Since you started the study, how have your asthma symptoms changed?” (0 = “Much better,” 1 = “A little better,” 2 = “The same,” 3 = “A little worse,” 4 = “Much worse”). The PGIC was completed by all child participants during the second study visit (EOS). Lastly, the CGIS measures the caregiver’s rating of their child’s current asthma symptom severity, and the CGIC assesses change in asthma symptom severity from the caregiver perspective.

### Statistical analysis

All analyses were performed using SAS version 9.4 except for the confirmatory factor analyses (CFAs), which were conducted using MPlus. All statistical tests were two-tailed with an alpha of 0.01, unless otherwise noted. The PAQLQ(S), ACQ-5, ACQ-IA-5, ACQ-IA-6, and C-ACT measures were all hand-entered, with appropriate quality assurance. All were scored according to the developers’ guidelines. There were no missing responses to the PGIS, PGIC, CGIS, or CGIC.

#### Descriptive statistics

Descriptive statistics for the ePASD were tabulated at all timepoints for the overall sample as well as for the pre- and post-pause samples. Response frequency distributions for each ePASD item were tabulated at Days 1, 2, 7, and 8. Descriptive statistics, including reported missing data, were also tabulated at Day 1 for the ACQ-5, ACQ-IA-5, ACQ-IA-6, PAQLQ(S), C-ACT, and global items (PGIS and CGIS).

#### ePASD structure

**Fig. 1:**
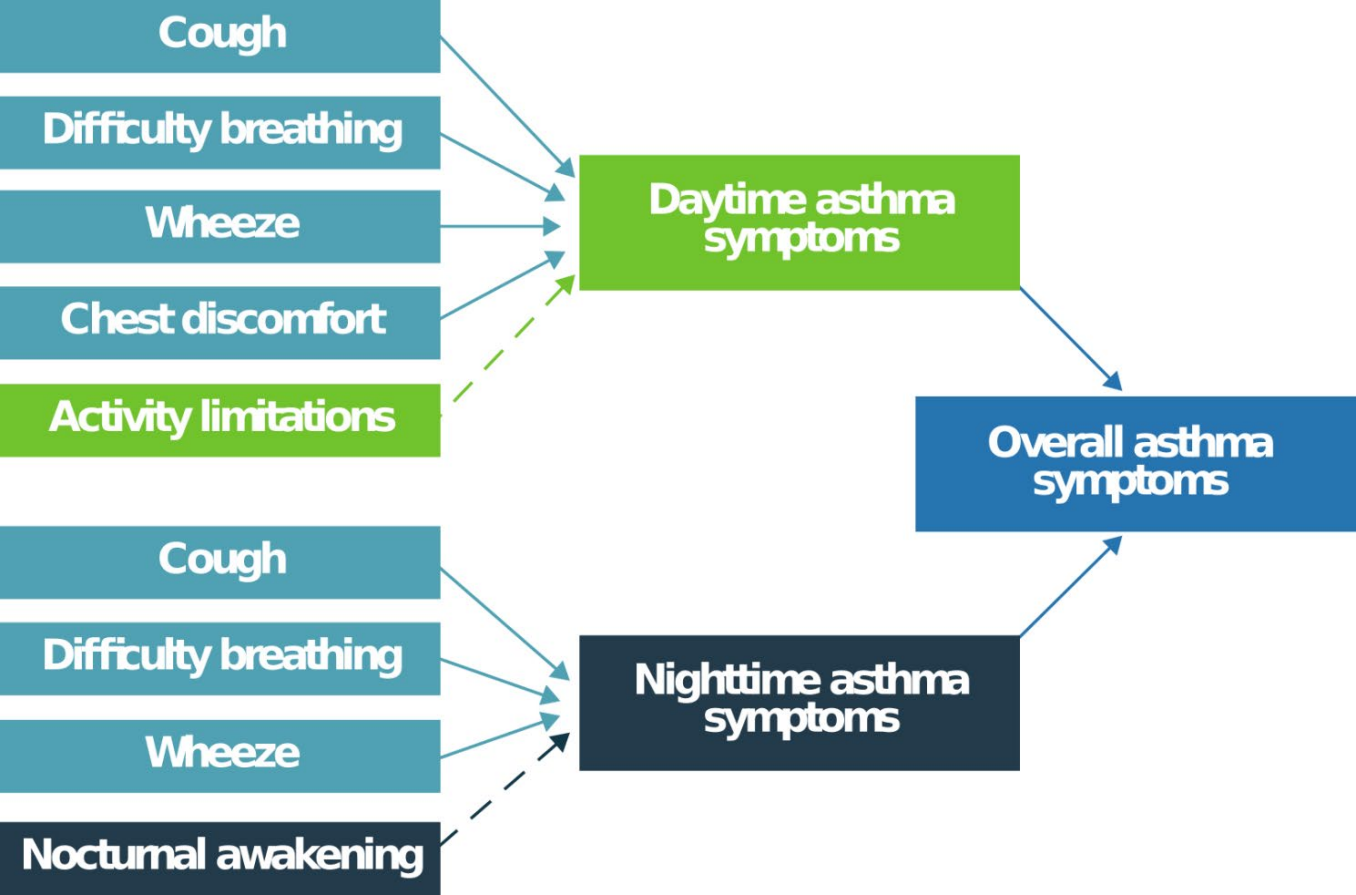
ePASD conceptual framework. ePASD = electronic Pediatric Asthma Symptom Diary. Note. This figure has been reproduced under a Creative Commons Attribution 4.0 license and is credited to Clark et al. Development and content validation of a self-completed, electronic Pediatric Asthma Symptom Diary. J Patient Rep Outcomes **6**, 25 (2022). 10.1186/s41687-022-00432-3

Inter-item correlations were computed using data from Days 1, 2, 7, and 8 to explore relationships among items. Factor analysis was performed using item-level data from the overall sample. The conceptual framework depicted in Fig. 1 was evaluated by fitting single-factor CFA models to the Day 1 and Day 8 item-level ePASD data. It is generally recommended to use a sample size larger than 200 and a minimum item-to-factor ratio of 3:1 for factor analysis [17]. However, in situations with a small number of factors (e.g., ≤ 8), high item-to-factor ratios (e.g., ≥ 6:1), and high communalities (e.g., standardized loadings ≥ 0.60), a minimum sample size of 100 may yield acceptable model fit [18, 19]. Note that the ePASD is described by a relatively simple conceptual framework, and each CFA model was a single factor model based on 3 to 7 items.

#### ePASD scoring

The optimal scoring of the ePASD was guided by the conceptual framework and the findings of the item-level analyses, CFAs, and internal consistency reliabilities. To support the internal consistency of ePASD composite scores, Cronbach’s coefficient alphas [20] were computed at all timepoints using the overall sample.

#### ePASD test-retest reliability

The test-retest reliability of the ePASD was assessed using the last 2 contiguous days of ePASD data collection as “test” and “retest.” The first analysis included only those participants whose EOS PGIC ratings were “The same” (PGIC = 2), whereas the second test-retest analysis included all participants. Weighted kappa coefficients were computed for the categorical ePASD items [12]. Intraclass correlation coefficients (ICCs) were calculated for ePASD composite scores; a two-way (subjects × time) mixed-effects analysis of variance (ANOVA) model with absolute agreement for single measures was used to compute ICC estimates of test-retest reliability [21–23].

#### ePASD validity

Correlational analyses were conducted (at Day 1 and EOS) using the overall sample to examine the construct validity of the ePASD items and composites. Correlations were examined for the expected patterns of relationships (specifically, the predicted sign and strength of the coefficients). Higher scores on the ePASD, ACQ-IA-5, ACQ-IA-6, PGIS, PGIC, CGIS, and CGIC indicate worse outcomes, such that positive correlations were predicted between these measures; lower scores on the PAQLQ(S) and C-ACT indicate worse outcomes, with negative correlations therefore predicted between the ePASD and the PAQLQ(S) and C-ACT. The strength of the correlations was assessed using Cohen’s criteria [24], where a correlation of at least 0.10 but less than 0.30 is small, a correlation of at least 0.30 but less than 0.50 is moderate, and a correlation of at least 0.50 is strong.

Specific item-level hypotheses included moderate to strong correlations between ePASD symptom items and PAQLQ(S) Symptoms scores, ACQ-IA-5 scores, ACQ-IA-6 scores, and PGIS and CGIS ratings; they also included moderate to strong correlations between ePASD activity items and PAQLQ(S) Activity Limitations scores. Longitudinal construct validity correlations similarly examined correlations between ePASD change scores and PAQLQ(S) change scores; ACQ-5, ACQ-IA-5, and ACQ-IA-6 change scores; and PGIC and CGIC ratings. Known-groups ANOVAs compared various subgroups of interest to provide evidence regarding the discriminating ability of the ePASD. For example, it was hypothesized that participants classified as “Mild” at screening would obtain better ePASD scores than those classified as “Severe,” as would participants classified as “Stable” at screening versus those classified as “Not stable.” Finally, using the overall sample and change from Day 1 to EOS Day − 1, the responsiveness of the ePASD was evaluated by computing effect-size estimates of change for each ePASD score.

## Results

### Descriptive statistics

Table 3 presents demographic and medical history characteristics for the overall sample (N = 91), the pre-pause sample (n = 24), and the post-pause all-virtual study sample (n = 67). All recruitment targets were reached, and the characteristics of the pre- and post-pause samples were generally very similar, with minor differences in race and ethnicity. For this reason, psychometric analysis results are presented for the overall (combined) sample. Nearly two-thirds of participants in the overall sample were male (n = 58; 63.7%), and slightly over half of the sample was White (n = 52; 57.1%). The average participant age was approximately 9.2 years (range, 6.0–11.0 years). As specifically targeted, 19 children (21.1%) in the overall sample were not able to read independently, while 71 children (78.9%) were reportedly able to read fluently. At screening, 86.8% of participants were “Stable” (n = 79), and there were at least 25 participants in each of the disease severity classifications (mild: n = 26, 28.6%; moderate: n = 40, 44.0%; and severe: n = 25, 27.5%). Day 1 descriptive statistics for the measures used to characterize asthma symptom severity (ACQ-5, ACQ-IA-5, ACQ-IA-6, PAQLQ[S], C-ACT, and global items) are presented in Table 4.

**Table 3:** Demographic and medical history characteristics

Characteristic	Overall N = 91	Pre-pause n = 24	Post-pause n = 67
**Participant age (years), mean (SD)**	9.2 (1.63)	9.1 (1.59)	9.2 (1.65)
Median; minimum, maximum	9.0; 6.0, 11.0	9.0; 6.0, 11.0	9.0; 6.0, 11.0
**Participant grade in school, n (%)**			
Kindergarten	5 (5.6)	1 (4.5)	4 (6.0)
1st grade	8 (9.0)	3 (13.6)	5 (7.5)
2nd grade	9 (10.1)	1 (4.5)	8 (11.9)
3rd grade	15 (16.9)	6 (27.3)	9 (13.4)
4th grade	21 (23.6)	5 (22.7)	16 (23.9)
5th grade	20 (22.5)	3 (13.6)	17 (25.4)
6th grade	11 (12.4)	3 (13.6)	8 (11.9)
**Participant reading skill**			
Unable to read	1 (1.1)	0 (0.0)	1 (1.5)
Able to read a few words	8 (8.9)	2 (8.3)	6 (9.1)
Reads full sentences with some difficulty	10 (11.1)	4 (16.7)	6 (9.1)
Reads fluently	71 (78.9)	18 (75.0)	53 (80.3)
**Participant gender, n (%)**			
Male	58 (63.7)	14 (58.3)	44 (65.7)
Female	33 (36.3)	10 (41.7)	23 (34.3)
**Caregiver gender, n (%)**			
Male	11 (12.1)	2 (8.3)	9 (13.4)
Female	79 (86.8)	22 (91.7)	57 (85.1)
**Participant race/ethnicity, n (%)**			
White	52 (57.1)	10 (41.7)	42 (62.7)
Black or African American	39 (42.9)	10 (41.7)	29 (43.3)
American Indian	1 (1.1)	0 (0.0)	1 (1.5)
Asian or Pacific Islander	5 (5.5)	1 (4.2)	4 (6.0)
Other	10 (11.0)	4 (16.7)	6 (9.0)
Hispanic or Latino	21 (23.1)	1 (4.2)	20 (29.9)
**Caregiver race/ethnicity, n (%)**			
White	55 (60.4)	12 (50.0)	43 (64.2)
Black or African American	31 (34.1)	10 (41.7)	21 (31.3)
American Indian	1 (1.1)	0 (0.0)	1 (1.5)
Asian or Pacific Islander	2 (2.2)	1 (4.2)	1 (1.5)
Other	4 (4.4)	1 (4.2)	3 (4.5)
Hispanic or Latino	9 (9.9)	0 (0.0)	9 (13.4)
**Participant asthma severity**			
Mild	26 (28.6)	13 (54.2)	13 (19.4)
Moderate	40 (44.0)	5 (20.8)	35 (52.2)
Severe	25 (27.5)	6 (25.0)	19 (28.4)
**Asthma symptoms in the last 2 weeks**			
Stable	79 (86.8)	18 (75.0)	61 (91.0)
Not stable	12 (13.2)	6 (25.0)	6 (9.0)
**Geographic location (site number)**			
Raleigh, North Carolina (1000)	22 (24.2)	22 (91.7)	0 (0.0)
Harrison, New York (2000)	24 (26.4)	2 (8.3)	22 (32.8)
Chalfont, Pennsylvania (3000)	23 (25.3)	0 (0.0)	23 (34.3)
Milwaukee, Wisconsin (4000)	22 (24.2)	0 (0.0)	22 (32.8)

SD = standard deviation

**Table 4:** Descriptive statistics for additional measures at day 1, overall sample

Day 1 COA score	n	Mean (SD)	Median	Min, max	Missing (%)
**ACQ-5**	6	1.40 (0.9)	1.6	0.0, 2.4	18 (75.0)
**ACQ-IA-5**	71	1.31 (1.1)	1.2	0.0, 4.6	20 (22.0)
**ACQ-IA-6**	71	1.27 (1.1)	1.2	0.0, 4.3	20 (22.0)
**C-ACT**	85	19.16 (3.8)	20.0	8.0, 26.0	6 (6.6)
**PAQLQ(S) Overall**	91	5.54 (1.3)	5.9	2.3, 7.0	0 (0.0)
**PAQLQ(S) Activity Limitation**	89	5.46 (1.2)	5.8	2.4, 7.0	2 (2.2)
**PAQLQ(S) Symptoms**	91	5.43 (1.3)	5.7	2.0, 7.0	0 (0.0)
**PAQLQ(S) Emotional Function**	91	5.73 (1.4)	6.3	1.9, 7.0	0 (0.0)
**PGIS**	88	0.44 (0.6)	0.0	0.0, 3.0	3 (3.3)
**CGIS**	86	0.67 (0.7)	1.0	0.0, 3.0	5 (5.5)

ACQ-5 = Asthma Control Questionnaire, Symptoms Only; ACQ-IA-5 = Asthma Control Questionnaire–Interviewer Administered, Symptoms Only; ACQ-IA-6 = Asthma Control Questionnaire–Interviewer Administered (including Item 6 Number of puffs); C-ACT = Childhood Asthma Control Test; CGIS = Caregiver Global Impression of Severity; COA = clinical outcome assessment; PAQLQ(S) = Pediatric Asthma Quality of Life Questionnaire–Standardized; PGIS = Patient Global Impression of Severity; SD = standard deviation

### ePASD item-level results

Item-level descriptive statistics for the overall sample are shown in Table S-1 (Supplementary Material 1). The average scores for all ePASD symptom items at all timepoints were less than 1 (“A little bad”) and in most cases close to 0 (e.g., “I didn’t cough”; “I didn’t wheeze”). Of the 4 daytime symptom items, Item D1 (Cough) achieved the highest mean values at most timepoints, with values ranging from 0.39 (SD = 0.6; n = 51) at Day 10 to 0.77 (SD = 0.9; n = 73) at Day 5. For days with adequate sample sizes (i.e., n > 20), Item D2 (Daytime Wheeze) generally obtained the smallest mean value, ranging from 0.22 (SD = 0.5, n = 73) at Day 7 to 0.45 (SD = 0.7, n = 82) at Day 4. Item N2 (Nighttime Wheeze) had the smallest mean scores of the nighttime symptom items, ranging from 0.16 (SD = 0.4, n = 51) at Day 9 to 0.48 (SD = 0.7, n = 82) at Day 3, at timepoints with adequate sample sizes. Median values were 0 or 1 for all daytime and nighttime symptom items. Average RFDs, which indicate the proportion of participants who reported an RFD, ranged from 0.43 at Day 3 (n = 67) to 0.60 at Day 11 (n = 10). That is, 43% of child participants reported using no rescue medication on Day 3, and 60% reported using no rescue medication on Day 11.

The ePASD items displayed satisfactory item-level test-retest reliability (Table S-2, Supplementary Material 1) and acceptable construct validity. There were very strong inter-item correlations at select timepoints but no consistent indications of item-level redundancies (Table S-3, Supplementary Material). As anticipated, we found positive correlations between the ePASD items and the ACQ-IA-5, ACQ-IA-6, PGIS, and CGIS and negative correlations between the ePASD items and the PAQLQ(S) and C-ACT (Table S-4, Supplemental Material). There was insufficient change in the present observational study to provide strong support for item-level responsiveness (Table S-5, Supplementary Material).

### ePASD structure

The CFAs conducted to confirm the structure underlying the ePASD are presented in Table 5. All item factor loadings were at least moderate in size for the ePASD scores analyzed: Daytime score (including Activity limitations; see Fig. 1), Daytime Symptoms score (not including Activity limitations), Nighttime score (including Nocturnal awakening), Nighttime Symptoms score (not including Nocturnal awakening), and Overall Symptoms score (not including Activity limitations or Nocturnal awakening). Due to the small sample sizes, the CFAs provided generally mixed support for the ePASD composite scores, but the model-based statistics indicated acceptable model fit for the Daytime score, Daytime Symptoms score, Nighttime score, and Overall Symptoms score. The Cronbach’s alphas [20] indicate item sets that are strongly related and capable of supporting a unidimensional scoring structure but are not redundant (Table 6).

**Table 5:** Single-factor CFA loadings

ePASD item	Daytime score (SE)	Daytime Symptoms score (SE)	Nighttime score (SE)	Nighttime Symptoms score (SE)^a^	Overall Symptoms score (SE)
**Day 1**	n = 82	n = 82	n = 82	n = 82	n = 89
D1 Cough	0.66 (0.07)	0.67 (0.08)	—	—	0.74 (0.06)
D2 Wheeze	0.73 (0.09)	0.78 (0.11)	—	—	0.72 (0.10)
D3 Chest	0.67 (0.07)	0.56 (0.09)	—	—	0.55 (0.08)
D4 Breathing	0.75 (0.07)	0.80 (0.09)	—	—	0.77 (0.07)
D5 Activities	0.73 (0.08)	—	—	—	—
N1 Cough	—	—	0.76 (0.11)	0.75 (0.14)	0.71 (0.09)
N2 Wheeze	—	—	0.62 (0.10)	0.68 (0.12)	0.77 (0.09)
N3 Breathing	—	—	0.90 (0.09)	0.86 (0.14)	0.79 (0.07)
N4 Wakening	—	—	0.91 (0.07)	—	—
CFI	0.97	1.00	1.00	—^a^	1.00
NFI	0.95	1.00	1.00	—^a^	1.00
RMSEA	0.12	0.0	0.0	—^a^	0.0
Chi-square statistic (df), *P* value	10.781 (5), 0.0559	1.626 (2), 0.4435	0.745 (2), 0.6889	—^a^ (0), —	12.036 (14), 0.6034
**Day 8**	n = 71	n = 71	n = 66	n = 66	n = 89
D1 Cough	0.68 (0.07)	0.65 (0.07)	—	—	0.72 (0.06)
D2 Wheeze	0.91 (0.04)	0.97 (0.04)	—	—	0.92 (0.04)
D3 Chest	0.71 (0.08)	0.75 (0.08)	—	—	0.70 (0.08)
D4 Breathing	0.93 (0.05)	0.88 (0.05)	—	—	0.88 (0.05)
D5 Activities	0.75 (0.07)	—	—	—	—
N1 Cough	—	—	0.81 (0.09)	0.76 (0.12)	0.75 (0.07)
N2 Wheeze	—	—	0.76 (0.10)	0.85 (0.11)	0.76 (0.08)
N3 Breathing	—	—	0.77 (0.09)	0.76 (0.11)	0.72 (0.09)
N4 Wakening	—	—	0.88 (0.10)	—	—
CFI	0.99	1.00	1.00	—^a^	0.95
NFI	0.98	1.00	0.99	—^a^	0.93
RMSEA	0.12	0.0	0.08	—^a^	0.14
Chi-square statistic (df), *P* value	10.138 (5), 0.0714	0.407 (2), 0.8158	2.775 (2), 0.2497	—^a^ (0), —	34.785 (14), 0.0016

CFA = confirmatory factor analysis; CFI = comparative fit index; df = degrees of freedom; ePASD = electronic Pediatric Asthma Symptom Diary; NFI = non-normed fit index; RMSEA = root mean square error of approximation; SE = standard error

^a^ The goodness of fit of the Nighttime Symptoms score cannot be determined because df = 0

**Table 6:** Internal consistency reliability (Cronbach’s Alphas)

ePASD score	Daytime Symptoms score alpha (n)	Daytime score alpha (n)	Nighttime Symptoms score alpha (n)	Nighttime score alpha (n)	Overall Symptoms score alpha (n)
Day 1	0.66 (82)	0.72 (65)	0.68 (81)	0.76 (81)	0.80 (74)
Day 2	0.78 (79)	0.87 (64)	0.73 (77)	0.78 (77)	0.80 (69)
Day 3	0.78 (73)	0.88 (56)	0.85 (82)	0.86 (82)	0.88 (67)
Day 4	0.85 (82)	0.89 (68)	0.87 (79)	0.88 (79)	0.91 (74)
Day 5	0.83 (73)	0.88 (66)	0.81 (78)	0.81 (78)	0.83 (67)
Day 6	0.81 (77)	0.87 (68)	0.77 (75)	0.82 (75)	0.89 (63)
Day 7	0.68 (73)	0.76 (60)	0.78 (77)	0.81 (77)	0.80 (65)
Day 8	0.79 (71)	0.80 (55)	0.68 (66)	0.75 (66)	0.81 (55)
Day 9	0.68 (61)	0.75 (48)	0.52 (51)	0.52 (51)	0.65 (43)
Day 10	0.68 (51)	0.78 (41)	0.59 (14)	0.74 (14)	0.83 (14)

ePASD = electronic Pediatric Asthma Symptom Diary

### ePASD scoring

**Fig. 2:**
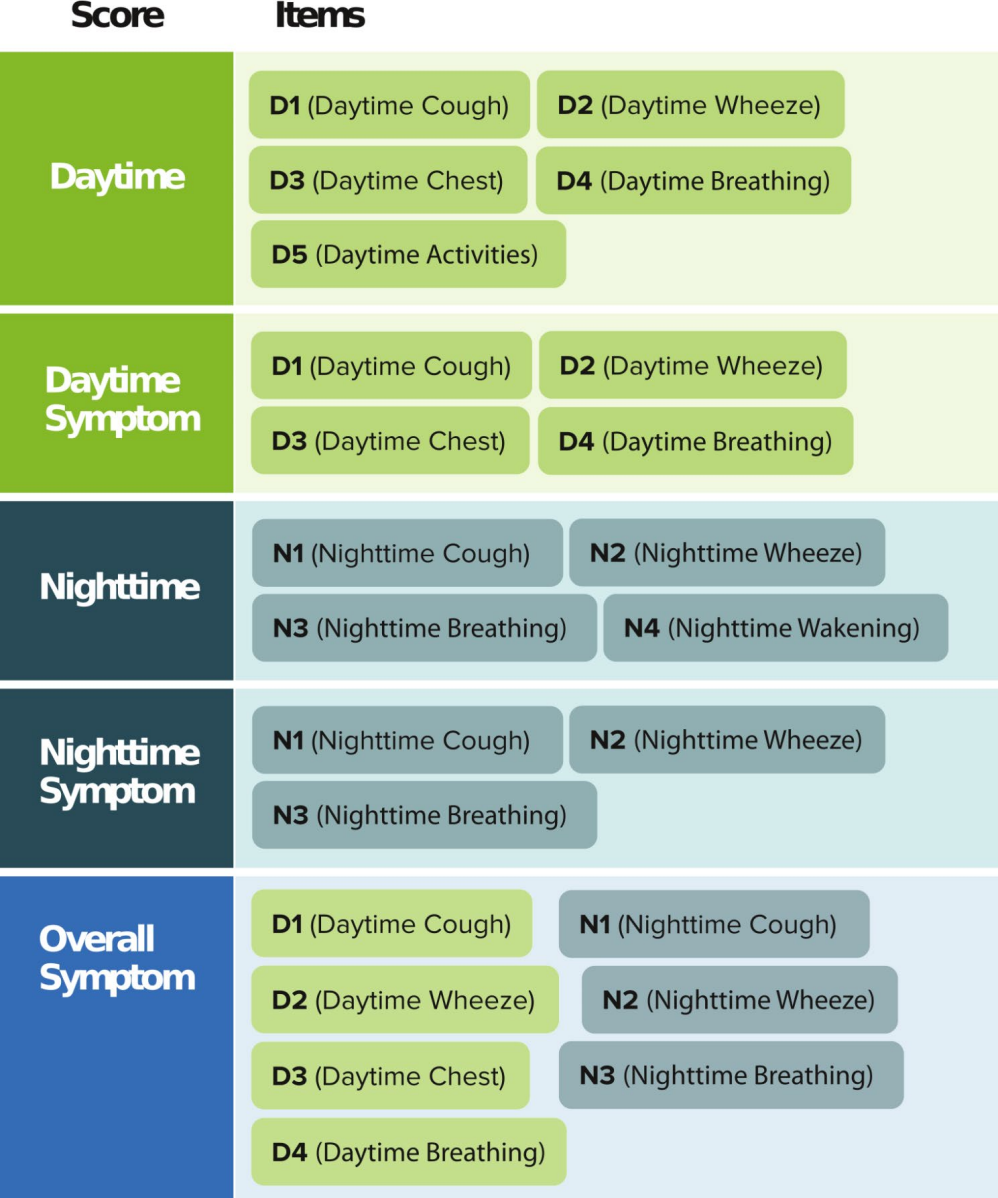
ePASD composite scores

The item-level analyses and CFA results indicated that 5 ePASD composite scores were reasonable candidates for further evaluation: Daytime Symptom score; Daytime score; Nighttime Symptom score; Nighttime score; and Overall Symptom score. The 5 ePASD composite scores were constructed as item averages, as described in Fig. 2. The ePASD composite scores use a 4-point response scale ranging from 0 to 3, with higher scores reflecting worse symptoms and impacts. In addition, 2 weekly scores were created to characterize rescue medication use. The number of RFDs was a count of the RFDs during any 7-day period of complete ePASD data, ranging from 0 to 7 (if a child did not have 7 days of ePASD data, their number of RFDs value was considered missing). RFD-Proportion was computed as the percentage of days in a 7-day period that were rescue medication free (4 or more days of ePASD data were required; if a child did not have 4 days of ePASD data, their RFD-Proportion was set to missing).

### ePASD composite-level results

The candidate ePASD scores were further evaluated with respect to reliability, construct validity, known-groups validity, and responsiveness. The average scores were close to 0 for all ePASD composite scores as well as for change in composite scores from Day 1 to EOS Day − 1 (Table 7).

**Table 7:** Descriptive statistics for ePASD composite scores, overall

ePASD composite score	Mean (SD), n	Median; min, max	Missing (%)
Day 1			
Daytime Symptom score	0.44 (0.4), 82	0.3; 0, 1.8	9 (9.9)
Daytime score	0.45 (0.4), 82	0.4; 0, 1.7	9 (9.9)
Nighttime Symptom score	0.42 (0.4), 82	0.3; 0, 1.7	9 (9.9)
Nighttime score	0.37 (0.4), 82	0.3; 0, 1.5	9 (9.9)
Overall Symptom score	0.43 (0.4), 82	0.3; 0, 1.3	9 (9.9)
**Change from Day 1 to EOS Day − 1**			
Daytime Symptom score	0.06 (0.4), 62	0.0; −1.0, 1.5	29 (31.9)
Daytime score	0.06 (0.4), 62	0.0; −1.1, 1.4	29 (31.9)
Nighttime Symptom score	−0.00 (0.4), 62	0.0; −1.0, 1.0	29 (31.9)
Nighttime score	−0.01 (0.4), 62	0.0; −1.0, 1.0	29 (31.9)
Overall Symptom score	0.03 (0.4), 62	0.0; −0.9, 1.0	29 (31.9)
**Weekly Scores**			
Number of RFDs	3.47 (3.0), 53	3.0; 0, 7.0	38 (41.8)
RFD-Proportion	0.52 (0.4), 70	0.5; 0, 1.0	21 (23.1)

EOS = end of study; ePASD = electronic Pediatric Asthma Symptom Diary; RFD = rescue medication–free day; SD = standard deviation

Notes: A study day is defined as the daytime item responses (Items D1-D6) followed by the nighttime item responses (Items N1-N5). For each study day, the daytime items are completed at the end of the day and the nighttime items are completed the next morning

#### Test-retest reliability

The test-retest stability of each ePASD composite score was evaluated using ePASD data at EOS Day − 1 and EOS Day − 2 for participants whose EOS PGIC rating was “The same” (PGIC = 2; n = 42–44) (Table 8). Using this subset of participants, only the ICC for the Overall Symptom score exceeded 0.70, the generally recommended minimum test-retest ICC for multi-item scales. A second analysis of all participants with data at EOS Day − 1 and EOS Day − 2 (n = 61) yielded higher test-retest ICCs, although the reliabilities for the Daytime Symptoms score and the Daytime score did not achieve the 0.70 criterion in this analysis.

**Table 8:** Test-retest reliability: ePASD composite scores

ePASD score	EOS Day −2 to EOS Day −1 ICC (95% CI), n PGIC = “The Same” (2)	EOS Day −2 to EOS Day −1 ICC (95% CI), n
Daytime Symptom score	0.60 (0.29, 0.80), 44	0.63 (0.39, 0.80), 61
Daytime score	0.60 (0.29, 0.80), 44	0.64 (0.40, 0.80), 61
Nighttime Symptom score	0.66 (0.36, 0.83), 42	0.73 (0.53, 0.85), 61
Nighttime score	0.65 (0.35, 0.83), 42	0.70 (0.49, 0.84), 61
Overall Symptom score	0.71 (0.44, 0.86), 44	0.73 (0.53, 0.85), 61

CI = confidence interval; EOS = end of study; ePASD = electronic Pediatric Asthma Symptom Diary; ICC = intraclass correlation coefficient; PGIC = Patient Global Impression of Change

#### Construct validity

Table 9 presents the construct validity of the ePASD scores as demonstrated through correlations with other participant- and caregiver-reported measures completed at Day 1 and EOS. As hypothesized, most of the correlations between the ePASD composites and the PGIS were moderate in size (0.30 ≤ r ≤ 0.49), with the exception of the 0.28 correlation with the Daytime Symptom score at Day 1 and the 0.27 correlation with the Nighttime Symptom score at EOS. As hypothesized, the ePASD composite scores correlated relatively strongly with the ACQ-IA-5, ACQ-IA-6, and C-ACT. It was hypothesized that the ePASD composite scores would correlate relatively strongly with PAQLQ(S) Symptoms and moderately with the more distal PAQLQ(S) scores. It was further expected the ePASD symptoms composites would correlate more strongly with the PAQLQ(S) Symptoms scores than with the ePASD Daytime score and Nighttime score. At EOS, all of the ePASD correlations with the PAQLQ(S) were moderate (0.30 ≤ r ≤ 0.49) or large (r > 0.50) in size, and at Day 1 most of the ePASD correlations with the PAQLQ(S) were large. The correlations between the ePASD composites and PAQLQ(S) Symptoms scores were larger than the correlations with the other PAQLQ(S) scores at EOS, as predicted; however, we did not observe this pattern at Day 1. Furthermore, the Daytime Symptoms scores and Nighttime Symptoms scores did not correlate more strongly with PAQLQ(S) Symptoms scores compared with the ePASD Daytime scores and Nighttime scores.

**Table 9:** Composite-level construct validity correlations

ePASD score	ACQ-IA-5	ACQ-IA-6	C-ACT	PAQLQ(S) Overall	PAQLQ(S) Activity Limitation	PAQLQ(S) Symptoms	PAQLQ(S) Emotional Function	PGIS	CGIS
**Day 1 (n = 39–82)**									
Daytime Symptom score	0.42*	0.44*	−0.43*	−0.61*	−0.59*	−0.58*	−0.60*	**0.28**	0.28
Daytime score	0.46*	0.48*	−0.40*	−0.65*	−0.64*	−0.61*	−0.63*	**0.31**	0.34*
Nighttime Symptom score	0.47*	0.49*	−0.48*	−0.57*	−0.58*	−0.57*	−0.49*	0.45*	0.35*
Nighttime score	0.49*	0.51*	−0.52*	−0.55*	−0.55*	−0.57*	−0.47*	0.48*	0.36*
Overall Symptom score	0.48*	0.50*	−0.49*	−0.64*	−0.64*	−0.62*	−0.60*	0.38*	0.34*
Number of RFDs	−0.69*	−0.74*	0.36	0.48*	0.46*	0.47*	0.46*	−0.16	−0.31
RFD-Proportion	−0.60*	−0.65*	0.33*	0.46*	0.39*	0.48*	0.42*	−0.07	−0.26
**EOS Day − 1 (n = 40–70)**									
Daytime Symptom score	0.42*	0.43*	−0.47*	−0.48*	−0.46*	−0.53*	−0.39*	0.41*	0.33
Daytime score	0.50*	0.51*	−0.45*	−0.51*	−0.51*	−0.55*	−0.42*	0.41*	0.34*
Nighttime Symptom score	0.59*	0.59*	−0.33*	−0.42*	−0.40*	−0.46*	−0.33*	**0.27**	0.40*
Nighttime score	0.60*	0.60*	−0.36*	−0.43*	−0.42*	−0.47*	−0.35*	**0.30**	0.40*
Overall Symptom score	0.48*	0.49*	−0.45*	−0.48*	−0.45*	−0.53*	−0.37*	0.38*	0.37*
Number of RFDs	−0.65*	−0.71*	0.37	0.43*	0.48*	0.45*	0.35	−0.48	−0.41
RFD-Proportion	−0.65*	−0.69*	0.43*	0.46*	0.48*	0.49*	0.37*	−0.48*	−0.41*

* P < 0.01

ACQ-IA-5 = Asthma Control Questionnaire–Interviewer Administered, Symptoms Only; ACQ-IA-6 = Asthma Control Questionnaire–Interviewer Administered (including Item 6 Number of puffs); C-ACT = Childhood Asthma Control Test; CGIS = Caregiver Global Impression of Severity; EOS = end of study; ePASD = electronic Pediatric Asthma Symptom Diary; PAQLQ(S) = Pediatric Asthma Quality of Life Questionnaire–Standardized; PGIS = Patient Global Impression of Severity; RFD = rescue medication–free day

Notes: EOS Day − 1 ePASD data were correlated with EOS PAQLQ(S), C-ACT, ACQ-IA-5, ACQ-IA-6, PGIS, and CGIS data. Correlation coefficients in **bold** were hypothesized to be relatively strong

Table 10 presents longitudinal construct validity correlations computed between changes in ePASD composite scores and the PGIC and CGIC ratings, as well as between changes in ePASD composites and the ACQ-IA-5, ACQ-IA-6, C-ACT, PAQLQ(S), PGIS, and CGIS. Most of the change correlations were trivial (r < 0.10) and small (0.10 ≤ r ≤ 0.29) in size. The correlations between the ePASD composites and the ACQ-IA and PAQLQ(S) scores were essentially 0 (*P* > 0.01); of the 3 PAQLQ(S) scores, the ePASD composite scores correlated slightly better with the PAQLQ(S) Symptoms scores. Change correlations were small or trivial between the ePASD composites and the PGIS and CGIS, but ePASD composite scores were moderately correlated with PGIC ratings.

**Table 10:** Composite-level longitudinal construct validity correlations

ePASD score	ACQ-IA-5	ACQ-IA-6	C-ACT	PAQLQ(S) Overall	PAQLQ(S) Activity Limitation	PAQLQ(S) Symptoms	PAQLQ(S) Emotional Function	PGIS	CGIS	PGIC	CGIC
**Change from Day 1 to EOS Day − 1 (n = 30–62)**											
Daytime Symptom score	**−0.04**	**−0.02**	**−0.31**	**−0.09**	0.05	**−0.16**	−0.07	**0.06**	−0.09	**0.33**	0.14
Daytime score	**0.10**	**0.11**	**−0.27**	**−0.12**	−0.00	**−0.20**	−0.04	**0.08**	−0.05	**0.32**	0.14
Nighttime Symptom score	**0.10**	**0.09**	**−0.03**	**−0.16**	−0.06	**−0.19**	−0.04	**0.02**	0.22	0.46*	0.32
Nighttime score	**0.11**	**0.09**	**−0.03**	**−0.13**	−0.05	**−0.16**	−0.03	**−0.00**	0.18	0.42*	0.30
Overall Symptom score	**−0.08**	**−0.07**	**−0.21**	**−0.11**	0.02	**−0.16**	−0.08	**−0.01**	−0.03	0.37*	0.17

* *P* < 0.01

ACQ-IA-5 = Asthma Control Questionnaire–Interviewer Administered, Symptoms Only; ACQ-IA-6 = Asthma Control Questionnaire–Interviewer Administered (including Item 6 Number of puffs); C-ACT = Childhood Asthma Control Test; CGIC = Caregiver Global Impression of Change; CGIS = Caregiver Global Impression of Severity; EOS = end of study; ePASD = electronic Pediatric Asthma Symptom Diary; PAQLQ(S) = Pediatric Asthma Quality of Life Questionnaire–Standardized; PGIC = Patient Global Impression of Change; PGIS = Patient Global Impression of Severity

Notes: ePASD change was computed using study Day 1 and EOS Day − 1 data; Day 1 and EOS data were used to compute change for the PAQLQ(S), CACT, ACQ-IA-5, ACQ-IA-6, PGIS, CGIS, PGIC, and CGIC. Correlation coefficients in **bold** were hypothesized to be relatively strong

#### Known-groups validity

Known-groups analyses provided solid support for the discriminating ability of the ePASD scores. It was hypothesized that participants classified as “Mild” at screening would have better Day 1 ePASD scores compared with participants who were classified as “Severe” at screening. All subgroup differences were in the hypothesized direction, and the hypothesis tests for the RFD variables achieved statistical significance (Table S-6 Supplementary Material). It was further hypothesized that participants classified as “Stable” at screening would have better Day 1 ePASD scores compared with participants classified as “Not stable.” Although all subgroup differences were in the hypothesized direction, none of the differences were statistically significant (Table S-6, Supplementary Material). Furthermore, participants with C-ACT scores < 20 had worse ePASD scores compared with participants with C-ACT scores ≥ 20 at both Day 1 and EOS Day − 1, as hypothesized. At Day 1, the subgroup differences were statistically significant for the Daytime Symptom score, Nighttime Symptom score, Nighttime score, and Overall Symptom score. At EOS Day − 1, the subgroup differences were statistically significant for the Daytime Symptom score, Daytime score, Nighttime score, Overall Symptom score, and RFD-Proportion.

#### Responsiveness

The effect-size estimates of change for each ePASD composite score, the observed score changes from Day 1 to EOS Day − 1, and t-tests are presented in Table 11. All of the effect sizes were small, and none of the observed score changes or t-tests were statistically significant.

**Table 11:** Responsiveness, Day 1 to EOS Day − 1

ePASD score	Effect-size estimate	Observed score change (SD), t *(P* value*)*
Daytime Symptom score	0.13	0.06 (0.45), − 0.99 (0.3262)
Daytime score	0.15	0.06 (0.43), − 1.14 (0.2608)
Nighttime Symptom score	−0.01	−0.00 (0.41), 0.05 (0.9590)
Nighttime score	−0.01	−0.01 (0.37), 0.11 (0.9104)
Overall Symptom score	0.08	0.03 (0.36), − 0.63 (0.5341)

EOS = end of study; ePASD = electronic Pediatric Asthma Symptom Diary; SD = standard deviation

## Discussion

The present study was conducted to evaluate the psychometric properties of the ePASD in children aged 6 to 11 years with mild to severe asthma. The ePASD is a novel, interactive, pediatric asthma PRO measure that facilitates self-completion in children [8] and was developed according to current FDA guidance [9, 11]. The results of this initial psychometric evaluation support the reliability and validity of the ePASD, as well as the planned context of use of the measure. The distributional characteristics, factor analyses, reliability estimates, and correlational and known-groups analyses provided important information supporting the use of the ePASD, as well as the ability of young children with asthma to self-report symptoms and impacts.

While the ISPOR good research practices report cites conflicting assessments of the ability of children aged 5–7 years to self-report, the task force authors note evidence of reliable and valid self-report in children aged as young as 5 years [10]. Consistent with this evidence, we report encouraging psychometric results in the present study, with children aged as young as 6 years being able to accurately self-report. Our findings also corroborate preliminary qualitative research that similarly found that children aged as young as 6 years were capable of providing reliable self-report [8], although the lack of control inherent in an observational study design makes it impossible to determine if and to what extent caregivers may have influenced children’s responses.

All the COAs included in the present study—the ePASD, ACQ-IA, C-ACT, PAQLQ(S), and global ratings—were in agreement that the children in the sample exhibited very few asthma-related symptoms and impacts at all timepoints, and consequently, demonstrated very little change over time. The lack of reported asthma symptoms and impacts observed in the present study may be related, in part, to lower exposure to asthma triggers due to social distancing, handwashing, mask mandates, and other directives, as this study began immediately prior to the COVID-19 global pandemic. Additionally, children may have had fewer opportunities to participate in activities, which may have influenced the ePASD impact scores. The present results must therefore be viewed through the lens of these extenuating circumstances, and future assessments should allow comparisons of self-reported asthma symptoms and impacts in nonpandemic environments. It is further possible that a longer observational study may have offered a better opportunity for study participants to demonstrate greater change in their asthma and, potentially, establish the responsiveness of the ePASD. Additionally, the PGIS and PGIC were developed for this study but not cognitively debriefed with children aged 6 to 11 years prior to their implementation. Although these items are simply worded and are similar to the ePASD, the recall period for the PGIS (i.e., right now) is shorter than that for the ePASD (i.e., last night or today), so the PGIS is less likely to have presented an issue for the child participants. It is possible that children’s misunderstanding of either of these items may have contributed to additional variability in the results.

Importantly, despite the restricted range of the responses in this sample, the ePASD items displayed satisfactory test-retest reliability and solid support for construct validity. However, because most participants were asymptomatic, further studies in the presence of more severe symptoms are needed. The internal consistency reliabilities were largely satisfactory for all ePASD composite scores, with minor exceptions. Additionally, composite-level test-retest reliabilities for the Nighttime Symptom score, Nighttime score, and Overall Symptom score were satisfactory, although those for the Daytime Symptom score and Daytime score were somewhat low. With respect to the construct validity, the pattern of convergent and divergent validity correlations supported the validity of the ePASD scores, as did the known-groups ANOVAs.

The lack of inclusion of participants with more severe asthma symptoms and impacts, the lack of reported asthma symptoms and impacts during the study, and the minimal change demonstrated by the ePASD items and the supportive COAs are important limitations of this study. Accordingly, we recommend a confirmatory evaluation of the ePASD composite scores, and further assessment to provide evidence for responsiveness to change. Future evaluation of the ePASD in the context of a clinical trial involving effective asthma treatment is necessary for the estimation of meaningful change, as well as the evaluation of longitudinal psychometric properties.

## Conclusion

To our knowledge, the ePASD is the first pediatric asthma PRO measure developed according to current FDA guidance that facilitates self-completion in children aged 6–11 years. The results of this initial psychometric evaluation indicate that the ePASD is a reliable and valid measure of asthma symptoms and proximal impacts in young children aged 6–11 years with mild, moderate, or severe asthma who may not be able to read independently. These results also describe the structure and scoring of the ePASD and lay the psychometric groundwork for the use of the ePASD in future clinical trials for the management of pediatric asthma. An ongoing pediatric asthma treatment trial is expected to recruit children with more severe symptoms and impacts and anticipated to provide further support for the validity and reliability of ePASD as well as evidence of the ePASD’s responsiveness to change.

### Electronic supplementary material

Below is the link to the electronic supplementary material.

Supplementary Material 1

## Data Availability

Most data generated or analyzed during this study are included in this published article and its supplementary information files.

## Abbreviations

ACQ: Asthma Control Questionnaire
ACQ-5: Asthma Control Questionnaire Symptoms Only
ACQ-IA: Asthma Control Questionnaire Interviewer Administered
ACQ-IA-5: Asthma Control Questionnaire Interviewer Administered; Symptoms Only
ACQ-IA-6: Asthma Control Questionnaire Interviewer Administered 6
ANOVA: Analysis of variance
C-ACT: Childhood Asthma Control Test
CFA: Confirmatory factor analysis
CGIC: Caregiver Global Impression of Change
CGIS: Caregiver Global Impression of Severity
COA: Clinical outcome assessment
COVID-19: Coronavirus disease 2019
EOS: End of study
ePASD: electronic Pediatric Asthma Symptom Diary
FDA: Food and Drug Administration
ICC: Intraclass correlation coefficient
PAQLQ: Pediatric Asthma Quality of Life Questionnaire–Standardized
PGIC: Patient Global Impression of Change
PGIS: Patient Global Impression of Severity
PRO: Patient-reported outcome
RFD: Rescue-medication‒free day
SD: Standard deviation
US: United States

## Declarations

### Previous presentation

Portions of this research were presented in poster format at the ISPOR Europe 2022 meeting from November 6–9, 2022.
