# Supplementary material for: Psychometric evaluation of an electronic Asthma Symptom Diary for young children
Source: J Patient Rep Outcomes. 2023 Oct 30;7:105. doi: 10.1186/s41687-023-00647-y (PMC10616038; doi:10.1186/s41687-023-00647-y)
Supplement: Supplementary file 1 — Supplementary Material 1 [file 41687_2023_647_MOESM1_ESM.docx]

**Supplementary Material**

**Table S-1. Item-Level Descriptive Statistics, Overall Sample**

| **ePASD item** | **n** | **Mean (SD)** | **Median** | **Min, max** | **Missing (%)** |
| --- | --- | --- | --- | --- | --- |
| **Day 1** |  |  |  |  |  |
| D1 Cough | 82 | 0.54 (0.6) | 0.0 | 0.0, 3.0 | 9 (9.9) |
| D2 Wheeze | 82 | 0.32 (0.5) | 0.0 | 0.0, 2.0 | 9 (9.9) |
| D3 Chest | 82 | 0.39 (0.6) | 0.0 | 0.0, 3.0 | 9 (9.9) |
| D4 Breathing | 82 | 0.50 (0.6) | 0.0 | 0.0, 2.0 | 9 (9.9) |
| D5a Activities (0-3) | 64 | 0.69 (0.8) | 1.0 | 0.0, 3.0 | 9 (12.3) |
| D5b Activities ^a^ | 18 | 1.94 (0.2) | 2.0 | 1.0, 2.0 | 9 (33.3) |
| D5 Activities (0-4) | 65 | 0.74 (0.9) | 1.0 | 0.0, 4.0 | 9 (12.2) |
| D6 Puffs | 82 | 1.04 (1.5) | 0.0 | 0.0, 8.0 | 9 (9.9) |
| N1 Cough | 81 | 0.49 (0.6) | 0.0 | 0.0, 2.0 | 10 (11.0) |
| N2 Wheeze | 82 | 0.27 (0.5) | 0.0 | 0.0, 2.0 | 9 (9.9) |
| N3 Breathing | 82 | 0.49 (0.6) | 0.0 | 0.0, 2.0 | 9 (9.9) |
| N4 Wakening | 82 | 0.24 (0.4) | 0.0 | 0.0, 1.0 | 9 (9.9) |
| N5 Puffs | 82 | 0.79 (1.3) | 0.0 | 0.0, 8.0 | 9 (9.9) |
| RFD | 75 | 0.48 (0.5) | 0.0 | 0.0, 1.0 | 16 (17.6) |
| **Day 2** |  |  |  |  |  |
| D1 Cough | 79 | 0.58 (0.6) | 1.0 | 0.0, 2.0 | 12 (13.2) |
| D2 Wheeze | 79 | 0.38 (0.5) | 0.0 | 0.0, 2.0 | 12 (13.2) |
| D3 Chest | 79 | 0.46 (0.7) | 0.0 | 0.0, 3.0 | 12 (13.2) |
| D4 Breathing | 79 | 0.54 (0.7) | 0.0 | 0.0, 3.0 | 12 (13.2) |
| D5a Activities (0-3) | 63 | 0.70 (0.8) | 0.0 | 0.0, 3.0 | 12 (16.0) |
| D5b Activities ^a^ | 16 | 1.94 (0.3) | 2.0 | 1.0, 2.0 | 12 (42.9) |
| D5 Activities (0-4) | 64 | 0.75 (0.9) | 0.5 | 0.0, 4.0 | 12 (15.8) |
| D6 Puffs | 79 | 0.91 (1.4) | 0.0 | 0.0, 6.0 | 12 (13.2) |
| N1 Cough | 77 | 0.64 (0.8) | 0.0 | 0.0, 3.0 | 14 (15.4) |
| N2 Wheeze | 77 | 0.43 (0.6) | 0.0 | 0.0, 3.0 | 14 (15.4) |
| N3 Breathing | 77 | 0.55 (0.7) | 0.0 | 0.0, 3.0 | 14 (15.4) |
| N4 Wakening | 77 | 0.25 (0.4) | 0.0 | 0.0, 1.0 | 14 (15.4) |
| N5 Puffs | 77 | 0.74 (1.2) | 0.0 | 0.0, 6.0 | 14 (15.4) |
| RFD | 69 | 0.52 (0.5) | 1.0 | 0.0, 1.0 | 22 (24.2) |
| **Day 3** |  |  |  |  |  |
| D1 Cough | 73 | 0.75 (0.8) | 1.0 | 0.0, 3.0 | 18 (19.8) |
| D2 Wheeze | 73 | 0.47 (0.7) | 0.0 | 0.0, 3.0 | 18 (19.8) |
| D3 Chest | 73 | 0.42 (0.6) | 0.0 | 0.0, 3.0 | 18 (19.8) |
| D4 Breathing | 73 | 0.59 (0.6) | 1.0 | 0.0, 2.0 | 18 (19.8) |
| D5a Activities (0-3) | 56 | 0.80 (0.9) | 1.0 | 0.0, 3.0 | 18 (24.3) |
| D5b Activities ^a^ | 17 | 2.00 (0.0) | 2.0 | 2.0, 2.0 | 18 (51.4) |
| D5 Activities (0-4) | 56 | 0.80 (0.9) | 1.0 | 0.0, 3.0 | 18 (24.3) |
| D6 Puffs | 73 | 1.12 (1.4) | 1.0 | 0.0, 6.0 | 18 (19.8) |
| N1 Cough | 82 | 0.62 (0.8) | 0.0 | 0.0, 3.0 | 9 (9.9) |
| N2 Wheeze | 82 | 0.48 (0.7) | 0.0 | 0.0, 3.0 | 9 (9.9) |
| N3 Breathing | 82 | 0.67 (0.7) | 1.0 | 0.0, 3.0 | 9 (9.9) |
| N4 Wakening | 82 | 0.23 (0.4) | 0.0 | 0.0, 1.0 | 9 (9.9) |
| N5 Puffs | 82 | 0.93 (1.4) | 0.0 | 0.0, 8.0 | 9 (9.9) |
| RFD | 67 | 0.43 (0.5) | 0.0 | 0.0, 1.0 | 24 (26.4) |
| **Day 4** |  |  |  |  |  |
| D1 Cough | 82 | 0.67 (0.9) | 0.0 | 0.0, 3.0 | 9 (9.9) |
| D2 Wheeze | 82 | 0.45 (0.7) | 0.0 | 0.0, 3.0 | 9 (9.9) |
| D3 Chest | 82 | 0.49 (0.8) | 0.0 | 0.0, 3.0 | 9 (9.9) |
| D4 Breathing | 82 | 0.59 (0.7) | 0.0 | 0.0, 3.0 | 9 (9.9) |
| D5a Activities (0-3) | 66 | 0.80 (1.0) | 0.5 | 0.0, 3.0 | 9 (12.0) |
| D5b Activities ^a^ | 16 | 1.88 (0.3) | 2.0 | 1.0, 2.0 | 9 (36.0) |
| D5 Activities (0-4) | 68 | 0.90 (1.1) | 1.0 | 0.0, 4.0 | 9 (11.7) |
| D6 Puffs | 82 | 1.26 (1.9) | 0.0 | 0.0, 8.0 | 9 (9.9) |
| N1 Cough | 79 | 0.57 (0.8) | 0.0 | 0.0, 3.0 | 12 (13.2) |
| N2 Wheeze | 79 | 0.27 (0.6) | 0.0 | 0.0, 3.0 | 12 (13.2) |
| N3 Breathing | 79 | 0.52 (0.8) | 0.0 | 0.0, 3.0 | 12 (13.2) |
| N4 Wakening | 79 | 0.18 (0.4) | 0.0 | 0.0, 1.0 | 12 (13.2) |
| N5 Puffs | 79 | 0.77 (1.4) | 0.0 | 0.0, 8.0 | 12 (13.2) |
| RFD | 74 | 0.53 (0.5) | 1.0 | 0.0, 1.0 | 17 (18.7) |
| **Day 5** |  |  |  |  |  |
| D1 Cough | 73 | 0.77 (0.9) | 1.0 | 0.0, 3.0 | 18 (19.8) |
| D2 Wheeze | 73 | 0.38 (0.6) | 0.0 | 0.0, 3.0 | 18 (19.8) |
| D3 Chest | 73 | 0.45 (0.8) | 0.0 | 0.0, 3.0 | 18 (19.8) |
| D4 Breathing | 73 | 0.53 (0.7) | 0.0 | 0.0, 3.0 | 18 (19.8) |
| D5a Activities (0-3) | 65 | 0.72 (0.8) | 1.0 | 0.0, 3.0 | 18 (21.7) |
| D5b Activities ^a^ | 8 | 1.88 (0.4) | 2.0 | 1.0, 2.0 | 18 (69.2) |
| D5 Activities (0-4) | 66 | 0.77 (0.9) | 1.0 | 0.0, 4.0 | 18 (21.4) |
| D6 Puffs | 73 | 1.23 (1.9) | 0.0 | 0.0, 8.0 | 18 (19.8) |
| N1 Cough | 78 | 0.60 (0.8) | 0.0 | 0.0, 3.0 | 13 (14.3) |
| N2 Wheeze | 78 | 0.36 (0.6) | 0.0 | 0.0, 2.0 | 13 (14.3) |
| N3 Breathing | 78 | 0.51 (0.7) | 0.0 | 0.0, 3.0 | 13 (14.3) |
| N4 Wakening | 78 | 0.24 (0.4) | 0.0 | 0.0, 1.0 | 13 (14.3) |
| N5 Puffs | 78 | 0.95 (1.4) | 0.0 | 0.0, 6.0 | 13 (14.3) |
| RFD | 67 | 0.45 (0.5) | 0.0 | 0.0, 1.0 | 24 (26.4) |
| **Day 6** |  |  |  |  |  |
| D1 Cough | 77 | 0.60 (0.7) | 1.0 | 0.0, 2.0 | 14 (15.4) |
| D2 Wheeze | 77 | 0.35 (0.7) | 0.0 | 0.0, 3.0 | 14 (15.4) |
| D3 Chest | 77 | 0.45 (0.7) | 0.0 | 0.0, 3.0 | 14 (15.4) |
| D4 Breathing | 77 | 0.53 (0.6) | 0.0 | 0.0, 2.0 | 14 (15.4) |
| D5a Activities (0-3) | 67 | 0.64 (0.8) | 1.0 | 0.0, 3.0 | 14 (17.3) |
| D5b Activities ^a^ | 10 | 1.90 (0.3) | 2.0 | 1.0, 2.0 | 14 (58.3) |
| D5 Activities (0-4) | 68 | 0.69 (0.9) | 1.0 | 0.0, 4.0 | 14 (17.1) |
| D6 Puffs | 77 | 1.19 (1.6) | 0.0 | 0.0, 8.0 | 14 (15.4) |
| N1 Cough | 75 | 0.59 (0.7) | 0.0 | 0.0, 3.0 | 16 (17.6) |
| N2 Wheeze | 75 | 0.31 (0.5) | 0.0 | 0.0, 2.0 | 16 (17.6) |
| N3 Breathing | 75 | 0.45 (0.6) | 0.0 | 0.0, 2.0 | 16 (17.6) |
| N4 Wakening | 75 | 0.24 (0.4) | 0.0 | 0.0, 1.0 | 16 (17.6) |
| N5 Puffs | 75 | 0.99 (1.5) | 0.0 | 0.0, 7.0 | 16 (17.6) |
| RFD | 63 | 0.48 (0.5) | 0.0 | 0.0, 1.0 | 28 (30.8) |
| **Day 7** |  |  |  |  |  |
| D1 Cough | 73 | 0.60 (0.7) | 0.0 | 0.0, 3.0 | 18 (19.8) |
| D2 Wheeze | 73 | 0.22 (0.5) | 0.0 | 0.0, 2.0 | 18 (19.8) |
| D3 Chest | 73 | 0.34 (0.7) | 0.0 | 0.0, 3.0 | 18 (19.8) |
| D4 Breathing | 73 | 0.40 (0.6) | 0.0 | 0.0, 2.0 | 18 (19.8) |
| D5a Activities (0-3) | 60 | 0.50 (0.7) | 0.0 | 0.0, 3.0 | 18 (23.1) |
| D5b Activities ^a^ | 13 | 2.00 (0.0) | 2.0 | 2.0, 2.0 | 18 (58.1) |
| D5 Activities (0-4) | 60 | 0.50 (0.7) | 0.0 | 0.0, 3.0 | 18 (23.1) |
| D6 Puffs | 73 | 0.68 (1.2) | 0.0 | 0.0, 6.0 | 18 (19.8) |
| N1 Cough | 77 | 0.48 (0.7) | 0.0 | 0.0, 3.0 | 14 (15.4) |
| N2 Wheeze | 77 | 0.25 (0.5) | 0.0 | 0.0, 2.0 | 14 (15.4) |
| N3 Breathing | 77 | 0.39 (0.7) | 0.0 | 0.0, 3.0 | 14 (15.4) |
| N4 Wakening | 77 | 0.18 (0.4) | 0.0 | 0.0, 1.0 | 14 (15.4) |
| N5 Puffs | 77 | 0.78 (1.3) | 0.0 | 0.0, 6.0 | 14 (15.4) |
| RFD | 65 | 0.52 (0.5) | 1.0 | 0.0, 1.0 | 26 (28.6) |
| **Day 8** |  |  |  |  |  |
| D1 Cough | 71 | 0.55 (0.7) | 0.0 | 0.0, 3.0 | 20 (22.0) |
| D2 Wheeze | 71 | 0.31 (0.6) | 0.0 | 0.0, 2.0 | 20 (22.0) |
| D3 Chest | 71 | 0.37 (0.6) | 0.0 | 0.0, 2.0 | 20 (22.0) |
| D4 Breathing | 71 | 0.46 (0.7) | 0.0 | 0.0, 2.0 | 20 (22.0) |
| D5a Activities (0-3) | 53 | 0.51 (0.8) | 0.0 | 0.0, 3.0 | 20 (27.4) |
| D5b Activities ^a^ | 18 | 1.89 (0.3) | 2.0 | 1.0, 2.0 | 20 (52.6) |
| D5 Activities (0-4) | 55 | 0.64 (1.0) | 0.0 | 0.0, 4.0 | 20 (26.7) |
| D6 Puffs | 71 | 1.04 (1.7) | 0.0 | 0.0, 8.0 | 20 (22.0) |
| N1 Cough | 66 | 0.42 (0.6) | 0.0 | 0.0, 3.0 | 25 (27.5) |
| N2 Wheeze | 66 | 0.24 (0.5) | 0.0 | 0.0, 2.0 | 25 (27.5) |
| N3 Breathing | 66 | 0.36 (0.6) | 0.0 | 0.0, 2.0 | 25 (27.5) |
| N4 Wakening | 66 | 0.23 (0.4) | 0.0 | 0.0, 1.0 | 25 (27.5) |
| N5 Puffs | 66 | 0.86 (1.3) | 0.0 | 0.0, 5.0 | 25 (27.5) |
| RFD | 55 | 0.58 (0.5) | 1.0 | 0.0, 1.0 | 36 (39.6) |
| **Day 9** |  |  |  |  |  |
| D1 Cough | 61 | 0.61 (0.7) | 1.0 | 0.0, 2.0 | 30 (33.0) |
| D2 Wheeze | 61 | 0.25 (0.4) | 0.0 | 0.0, 1.0 | 30 (33.0) |
| D3 Chest | 61 | 0.30 (0.5) | 0.0 | 0.0, 2.0 | 30 (33.0) |
| D4 Breathing | 61 | 0.56 (0.8) | 0.0 | 0.0, 3.0 | 30 (33.0) |
| D5a Activities (0-3) | 46 | 0.59 (0.7) | 0.0 | 0.0, 3.0 | 30 (39.5) |
| D5b Activities ^a^ | 15 | 1.87 (0.4) | 2.0 | 1.0, 2.0 | 30 (66.7) |
| D5 Activities (0-4) | 48 | 0.73 (1.0) | 0.0 | 0.0, 4.0 | 30 (38.5) |
| D6 Puffs | 61 | 1.10 (1.4) | 0.0 | 0.0, 6.0 | 30 (33.0) |
| N1 Cough | 51 | 0.31 (0.6) | 0.0 | 0.0, 2.0 | 40 (44.0) |
| N2 Wheeze | 51 | 0.16 (0.4) | 0.0 | 0.0, 2.0 | 40 (44.0) |
| N3 Breathing | 51 | 0.37 (0.5) | 0.0 | 0.0, 2.0 | 40 (44.0) |
| N4 Wakening | 51 | 0.16 (0.4) | 0.0 | 0.0, 1.0 | 40 (44.0) |
| N5 Puffs | 51 | 0.69 (1.1) | 0.0 | 0.0, 4.0 | 40 (44.0) |
| RFD | 43 | 0.51 (0.5) | 1.0 | 0.0, 1.0 | 48 (52.7) |
| **Day 10** |  |  |  |  |  |
| D1 Cough | 51 | 0.39 (0.6) | 0.0 | 0.0, 2.0 | 40 (44.0) |
| D2 Wheeze | 51 | 0.29 (0.5) | 0.0 | 0.0, 2.0 | 40 (44.0) |
| D3 Chest | 51 | 0.47 (0.8) | 0.0 | 0.0, 3.0 | 40 (44.0) |
| D4 Breathing | 51 | 0.41 (0.6) | 0.0 | 0.0, 2.0 | 40 (44.0) |
| D5a Activities (0-3) | 41 | 0.73 (1.0) | 0.0 | 0.0, 3.0 | 40 (49.4) |
| D5b Activities ^a^ | 10 | 2.00 (0.0) | 2.0 | 2.0, 2.0 | 40 (80.0) |
| D5 Activities (0-4) | 41 | 0.73 (1.0) | 0.0 | 0.0, 3.0 | 40 (49.4) |
| D6 Puffs | 51 | 1.00 (1.5) | 0.0 | 0.0, 7.0 | 40 (44.0) |
| N1 Cough | 14 | 0.50 (0.7) | 0.0 | 0.0, 2.0 | 77 (84.6) |
| N2 Wheeze | 14 | 0.14 (0.4) | 0.0 | 0.0, 1.0 | 77 (84.6) |
| N3 Breathing | 14 | 0.21 (0.4) | 0.0 | 0.0, 1.0 | 77 (84.6) |
| N4 Wakening | 14 | 0.29 (0.5) | 0.0 | 0.0, 1.0 | 77 (84.6) |
| N5 Puffs | 14 | 0.93 (1.7) | 0.0 | 0.0, 5.0 | 77 (84.6) |
| RFD | 14 | 0.57 (0.5) | 1.0 | 0.0, 1.0 | 77 (84.6) |
| **Day 11** |  |  |  |  |  |
| D1 Cough | 10 | 0.40 (0.5) | 0.0 | 0.0, 1.0 | 81 (89.0) |
| D2 Wheeze | 10 | 0.10 (0.3) | 0.0 | 0.0, 1.0 | 81 (89.0) |
| D3 Chest | 10 | 0.20 (0.4) | 0.0 | 0.0, 1.0 | 81 (89.0) |
| D4 Breathing | 10 | 0.10 (0.3) | 0.0 | 0.0, 1.0 | 81 (89.0) |
| D5a Activities (0-3) | 5 | 0.00 (0.0) | 0.0 | 0.0, 0.0 | 81 (94.2) |
| D5b Activities ^a^ | 5 | 2.00 (0.0) | 2.0 | 2.0, 2.0 | 81 (94.2) |
| D5 Activities (0-4) | 5 | 0.00 (0.0) | 0.0 | 0.0, 0.0 | 81 (94.2) |
| D6 Puffs | 10 | 1.20 (2.7) | 0.0 | 0.0, 8.0 | 81 (89.0) |
| N1 Cough | 10 | 0.30 (0.5) | 0.0 | 0.0, 1.0 | 81 (89.0) |
| N2 Wheeze | 10 | 0.30 (0.5) | 0.0 | 0.0, 1.0 | 81 (89.0) |
| N3 Breathing | 10 | 0.20 (0.4) | 0.0 | 0.0, 1.0 | 81 (89.0) |
| N4 Wakening | 10 | 0.20 (0.4) | 0.0 | 0.0, 1.0 | 81 (89.0) |
| N5 Puffs | 10 | 0.90 (1.4) | 0.0 | 0.0, 4.0 | 81 (89.0) |
| RFD | 10 | 0.60 (0.5) | 1.0 | 0.0, 1.0 | 81 (89.0) |
| **Day 12** |  |  |  |  |  |
| D1 Cough | 5 | 0.40 (0.5) | 0.0 | 0.0, 1.0 | 86 (94.5) |
| D2 Wheeze | 5 | 0.60 (0.5) | 1.0 | 0.0, 1.0 | 86 (94.5) |
| D3 Chest | 5 | 0.40 (0.5) | 0.0 | 0.0, 1.0 | 86 (94.5) |
| D4 Breathing | 5 | 0.60 (0.5) | 1.0 | 0.0, 1.0 | 86 (94.5) |
| D5a Activities (0-3) | 2 | 0.00 (0.0) | 0.0 | 0.0, 0.0 | 86 (97.7) |
| D5b Activities ^a^ | 3 | 2.00 (0.0) | 2.0 | 2.0, 2.0 | 86 (96.6) |
| D5 Activities (0-4) | 2 | 0.00 (0.0) | 0.0 | 0.0, 0.0 | 86 (97.7) |
| D6 Puffs | 5 | 1.40 (0.9) | 2.0 | 0.0, 2.0 | 86 (94.5) |
| RFD | 0 | — | — | — | 91 (100.0) |
| **Change from Day 1 to EOS Day −1** | | |  |  |  |
| D1 Cough | 62 | 0.18 (0.7) | 0.0 | −1.0, 2.0 | 29 (31.9) |
| D2 Wheeze | 62 | 0.06 (0.7) | 0.0 | −1.0, 3.0 | 29 (31.9) |
| D3 Chest | 62 | −0.05 (0.7) | 0.0 | −3.0, 2.0 | 29 (31.9) |
| D4 Breathing | 62 | 0.03 (0.8) | 0.0 | −1.0, 2.0 | 29 (31.9) |
| D5 Activities (0-4) | 39 | 0.10 (1.0) | 0.0 | −2.0, 4.0 | 52 (57.1) |
| D6 Puffs | 62 | −0.06 (1.5) | 0.0 | −5.0, 4.0 | 29 (31.9) |
| N1 Cough | 61 | 0.05 (0.7) | 0.0 | −1.0, 2.0 | 30 (33.0) |
| N2 Wheeze | 62 | −0.02 (0.4) | 0.0 | −1.0, 1.0 | 29 (31.9) |
| N3 Breathing | 62 | −0.03 (0.6) | 0.0 | −1.0, 2.0 | 29 (31.9) |
| N4 Wakening | 62 | −0.02 (0.4) | 0.0 | −1.0, 1.0 | 29 (31.9) |
| N5 Puffs | 62 | 0.08 (1.0) | 0.0 | −3.0, 3.0 | 29 (31.9) |

EOS = end of study; ePASD = electronic Pediatric Asthma Symptom Diary; RFD = rescue-medication‒free day; SD = standard deviation.

Notes: A study day is defined as the daytime item responses (D1-D6) followed by the nighttime item responses (N1‑N5). For each study day, the daytime items (D1-D6) are completed at the end of the day and the nighttime items (N1-N5) are completed the next morning.

^a^ Includes respondents who reported “I didn’t do any” to the D5a Activities item. Response options were: 1 = “I couldn’t because of my asthma” and 2 = “I just didn’t do any.”

**Table S-2. Item-Level Test-Retest Reliability**

| **ePASD score** | **EOS Day −2 to EOS Day −1 Kappa (95% CI), n PGIC = “The Same” (2)** | **EOS Day −2 to EOS Day −1 Kappa (95% CI), n** |
| --- | --- | --- |
| D1 Cough | 0.41 (0.12, 0.69), 44 | 0.50 (0.27, 0.72), 61 |
| D2 Wheeze | 0.50 (0.15, 0.85), 44 | 0.53 (0.21, 0.84), 61 |
| D3 Chest | 0.54 (0.25, 0.83), 44 | 0.59 (0.35, 0.83), 61 |
| D4 Breathing | 0.42 (0.14, 0.70), 44 | 0.49 (0.27, 0.70), 61 |
| D5 Activities | 0.42 (0.23, 0.62), 35 | 0.51 (0.33, 0.69), 46 |
| D6 Puffs | 0.70 (0.47, 0.94), 44 | 0.72 (0.52, 0.92), 61 |
| N1 Cough | 0.52 (0.25, 0.78), 42 | 0.61 (0.46, 0.77), 61 |
| N2 Wheeze | 0.34 (0.02, 0.66), 42 | 0.41 (0.14, 0.67), 61 |
| N3 Breathing | 0.73 (0.56, 0.89), 42 | 0.77 (0.64, 0.89), 61 |
| N4 Wakening | 0.44 (0.11, 0.76), 42 | 0.44 (0.18, 0.71), 61 |
| N5 Puffs | 0.85 (0.73, 0.97), 42 | 0.72 (0.51, 0.92), 61 |
| RFD | 0.89 (0.74, 1.00), 36 | 0.84 (0.69, 0.99), 51 |

EOS = end of study; ePASD = electronic Pediatric Asthma Symptom Diary; PGIC = Patient Global Impression of Change; RFD = rescue-medication‒free day.

Note: Simple kappas were computed for Item N4 (Wakening) and RFD; weighted kappas were computed for all other ePASD items.

**Table S-3. ePASD Inter-Item Correlations, Overall Sample**

| **ePASD item** | **D1** | **D2** | **D3** | **D4** | **D5** | **D6** | **N1** | **N2** | **N3** | **N4** | **N5** | **Item-total *r*** |
| --- | --- | --- | --- | --- | --- | --- | --- | --- | --- | --- | --- | --- |
| **Day 1 and Day 2 (n = 47 to 82) ^a^** | | | | |  |  |  |  |  |  |  |  |
| D1 Cough | 0.47* | 0.60* | 0.66* | 0.60* | 0.73* | 0.58* | 0.49* | 0.35 | 0.28 | 0.66* | 0.24 | 0.63 |
| D2 Wheeze | 0.49* | 0.50* | 0.45* | 0.60* | 0.58* | 0.55* | 0.35 | 0.79* | 0.46* | 0.61* | 0.28 | 0.62 |
| D3 Chest | 0.42* | 0.43* | 0.52* | 0.77* | 0.83* | 0.54* | 0.36 | 0.26 | 0.41 | 0.31 | 0.45* | 0.45 |
| D4 Breathing | 0.55* | 0.68* | 0.40* | 0.55* | 0.91* | 0.62* | 0.57* | 0.44* | 0.63* | 0.51* | 0.42* | 0.68 |
| D5 Activities ^c^ | 0.46* | 0.47* | 0.59* | 0.50* | 0.83* | 0.66* | 0.44* | 0.27 | 0.50* | 0.36 | 0.29 | 0.67 |
| D6 Puffs | 0.37 | 0.67* | 0.34 | 0.48* | 0.37 | 0.68* | 0.43* | 0.53* | 0.39 | 0.32 | 0.75* | 0.65 |
| N1 Cough | 0.62* | 0.40 | 0.35 | 0.36 | 0.50* | 0.30 | 0.60* | 0.48* | 0.63* | 0.79* | 0.36 | 0.52 |
| N2 Wheeze | 0.57* | 0.52* | 0.38 | 0.68* | 0.38 | 0.56* | 0.51* | 0.87* | 0.66* | 0.76* | 0.53* | 0.49 |
| N3 Breathing | 0.56* | 0.56* | 0.46* | 0.57* | 0.55* | 0.60* | 0.64* | 0.59* | 0.62* | 0.63* | 0.47* | 0.47 |
| N4 Wakening | 0.53* | 0.46 | 0.11 | 0.45 | 0.46 | 0.55* | 0.70* | 0.53* | 0.83* | 0.73* | 0.45 | 0.49 |
| N5 Puffs | 0.21 | 0.59* | 0.31 | 0.55* | 0.28 | 0.80* | 0.41* | 0.53* | 0.54* | 0.62* | 0.76* | 0.44 |
| Item-Total *r* | 0.42 | 0.71 | 0.36 | 0.63 | 0.49 | 0.61 | 0.45 | 0.56 | 0.70 | 0.58 | 0.60 | **—** |
| **Day 7 and Day 8 (n = 40 to 77) ^b^** | | | | |  |  |  |  |  |  |  |  |
| D1 Cough | 0.51* | 0.65* | 0.48* | 0.56* | 0.56* | 0.55* | 0.80* | 0.38 | 0.50* | 0.65* | 0.42 | 0.52 |
| D2 Wheeze | 0.42 | 0.47 | 0.71* | 0.85* | 0.59* | 0.58* | 0.39 | 0.69* | 0.47 | 0.46 | 0.49 | 0.56 |
| D3 Chest | 0.38 | 0.62* | 0.52* | 0.68* | 0.37 | 0.38 | 0.23 | 0.53* | 0.34 | 0.27 | 0.31 | 0.33 |
| D4 Breathing | 0.41* | 0.78* | 0.73* | 0.59* | 0.74* | 0.57* | 0.41 | 0.63* | 0.74* | 0.60* | 0.50* | 0.62 |
| D5 Activities ^c^ | 0.35 | 0.46 | 0.48* | 0.69* | 0.77* | 0.60* | 0.43 | 0.77* | 0.80* | 0.72* | 0.59* | 0.65 |
| D6 Puffs | 0.21 | 0.48* | 0.27 | 0.34 | 0.45 | 0.61* | 0.48 | 0.61* | 0.66* | 0.64* | 0.88* | 0.65 |
| N1 Cough | 0.55* | 0.56* | 0.13 | 0.44 | 0.51* | 0.32 | 0.83* | 0.65* | 0.58* | 0.74* | 0.59* | 0.60 |
| N2 Wheeze | 0.22 | 0.53 | 0.22 | 0.43 | 0.22 | 0.03 | 0.65* | 0.74* | 0.64* | 0.52 | 0.82* | 0.62 |
| N3 Breathing | 0.41 | 0.43 | 0.35 | 0.53* | 0.53* | 0.20 | 0.68* | 0.74* | 0.78* | 0.72* | 0.72* | 0.69 |
| N4 Wakening | 0.12 | 0.41 | 0.31 | 0.40 | 0.65* | 0.54* | 0.81* | 0.59* | 0.86* | 0.69* | 0.63* | 0.68 |
| N5 Puffs | 0.21 | 0.35 | 0.17 | 0.30 | 0.46 | 0.66* | 0.62* | 0.38 | 0.69* | 0.75* | 0.87* | 0.65 |
| Item-Total *r* | 0.47 | 0.56 | 0.38 | 0.61 | 0.64 | 0.48 | 0.67 | 0.44 | 0.63 | 0.60 | 0.57 | **—** |

* *P* < 0.01.

ePASD = electronic Pediatric Asthma Symptom Diary.

Note: The daytime symptom items are shaded in light orange and the nighttime symptom items are shaded in light blue.

^a^ In the upper panel, the Day 1 inter-item correlations are in the bottom left triangle below the main diagonal and the Day 2 inter-item correlations are in the top right triangle above the main diagonal; the correlations between Day 1 and Day 2 are underlined on the main diagonal.

^b^ In the lower panel, the Day 7 inter-item correlations are in the bottom left triangle below the main diagonal and the Day 8 inter-item correlations are in the top right triangle above the main diagonal; the correlations between Day 7 and Day 8 are underlined on the main diagonal.

^c^ D5 was constructed using D5a and D5b and rescaled for analysis purposes (0 = 0, 1 = 0.75, 2 = 1.5, 3 = 2.25, 4 = 3).

**Table S-4. Item-Level Construct Validity Correlations, Overall Sample**

| **ePASD item** | **ACQ-5** | **ACQ-IA-5** | **ACQ-IA-6** | **C-ACT** | **PAQLQ(S) Overall** | **PAQLQ(S) Activity Limitation** | **PAQLQ(S) Symptoms** | **PAQLQ(S) Emotional Function** | **PGIS** | **CGIS** |
| --- | --- | --- | --- | --- | --- | --- | --- | --- | --- | --- |
| **Day 1 (n = 2 to 82)** | |  |  |  |  |  |  |  |  |  |
| D1 Cough | 1.00^ | 0.48* | 0.47* | **−0.42*** | **−0.51*** | −0.51* | **−0.48*** | −0.50* | **0.29** | 0.14 |
| D2 Wheeze | **—** | **0.34** | **0.36** | **−0.46*** | **−0.50*** | −0.47* | **−0.48*** | −0.51* | **0.26** | 0.49* |
| D3 Chest | — | 0.22 | 0.27 | −0.20 | **−0.47*** | −0.52* | **−0.48*** | −0.41* | **0.19** | 0.22 |
| D4 Breathing | **—** | **0.44*** | **0.45*** | −0.39* | **−0.48*** | −0.43* | **−0.45*** | −0.49* | **0.31** | 0.22 |
| D5 Activities ^a^ | **1.00^** | **0.55*** | **0.58*** | **−0.19** | **−0.53*** | **−0.58*** | −0.49* | −0.50* | **0.41** | 0.55* |
| D6 Puffs | 0.39^ | 0.55* | 0.59* | −0.59* | **−0.51*** | −0.47* | −0.55* | −0.45* | **0.43*** | 0.42* |
| N1 Cough | — | 0.25 | 0.26 | **−0.31** | **−0.36*** | −0.39* | **−0.37*** | −0.29 | **0.36** | 0.27 |
| N2 Wheeze | **0.85^** | **0.61*** | **0.63*** | **−0.73*** | **−0.55*** | −0.56* | **−0.57*** | −0.49* | **0.58*** | 0.52* |
| N3 Breathing | **0.79^** | **0.52*** | **0.55*** | −0.36* | **−0.59*** | −0.62* | **−0.58*** | −0.51* | **0.45*** | 0.34 |
| N4 Wakening | **1.00^** | **0.58*** | **0.60*** | **−0.60*** | **−0.43*** | **−0.39*** | −0.50* | −0.33 | **0.59*** | 0.42 |
| N5 Puffs | −0.15^ | 0.60* | 0.65* | −0.48* | **−0.53*** | −0.47* | −0.55* | −0.49* | **0.31** | 0.41* |

* *P* < 0.01.

^ n < 20.

ACQ-5 = Asthma Control Questionnaire, Symptoms Only; ACQ-IA-5 = Asthma Control Questionnaire–Interviewer-Administered, Symptoms Only; ACQ-IA-6 = Asthma Control Questionnaire–Interviewer-Administered (including Item 6 Number of puffs); C-ACT = Childhood Asthma Control Test; CGIS = Caregiver Global Impression of Severity; EOS = end of study; ePASD = electronic Pediatric Asthma Symptom Diary; PAQLQ(S) = Pediatric Asthma Quality of Life Questionnaire–Standardized; PGIS = Patient Global Impression of Severity.

Notes: Day 1 ePASD data were correlated with EOS PAQLQ(S), C-ACT, ACQ-5, ACQ-IA-5, ACQ-IA-6, PGIS, and CGIS data. Correlation coefficients in **bold** are hypothesized to be relatively strong.

^a^ D5 was constructed using D5a and D5b and rescaled for analysis purposes (0 = 0, 1 = 0.75, 2 = 1.5, 3 = 2.25, 4 = 3).

**Table S-5. Item-Level Responsiveness**

| **ePASD score** | **Effect-size estimate** | **Observed score change (SD), t *(P* value*)*** |
| --- | --- | --- |
|  |  |  |
| D1 Cough | 0.29 | 0.18 (0.69), −2.02 (0.0473) |
| D2 Wheeze | 0.12 | 0.06 (0.72), −0.70 (0.4840) |
| D3 Chest | −0.08 | −0.05 (0.73), 0.52 (0.6055) |
| D4 Breathing | 0.05 | 0.03 (0.77), −0.33 (0.7418) |
| D5 Activities | 0.12 | 0.08 (0.75), −0.64 (0.5234) |
| D6 Puffs | −0.04 | −0.06 (1.49), 0.34 (0.7346) |
| N1 Cough | 0.08 | 0.05 (0.67), −0.57 (0.5680) |
| N2 Wheeze | −0.03 | −0.02 (0.38), 0.33 (0.7418) |
| N3 Breathing | −0.05 | −0.03 (0.63), 0.41 (0.6865) |
| N4 Wakening | −0.04 | −0.02 (0.42), 0.30 (0.7657) |
| N5 Puffs | 0.06 | 0.08 (1.03), −0.62 (0.5395) |

EOS = end of study; ePASD = electronic Pediatric Asthma Symptom Diary; SD = standard deviation.

**Table S-6. Known-Groups ANOVAs**

| **ePASD score** | **Subgroup 1** | | **Subgroup 2** | | ***F* statistic, *P* value** |
| --- | --- | --- | --- | --- | --- |
|  | **n** | **Mean (SD)** | **n** | **Mean (SD)** |  |
| **Screening severity** | **“Mild” at screening** | | **“Severe” at screening** | |  |
| Daytime Symptom score | 20 | 0.36 (0.4) | 24 | 0.48 (0.4) | 0.85, 0.3606 |
| Daytime score | 20 | 0.39 (0.4) | 24 | 0.48 (0.4) | 0.55, 0.4621 |
| Nighttime Symptom score | 23 | 0.35 (0.4) | 22 | 0.44 (0.4) | 0.54, 0.4669 |
| Nighttime score | 23 | 0.33 (0.4) | 22 | 0.38 (0.4) | 0.17, 0.6787 |
| Overall Symptom score | 20 | 0.37 (0.4) | 24 | 0.46 (0.3) | 0.77, 0.3858 |
| Number of RFDs | 11 | 5.55 (2.8) | 17 | 2.24 (2.8) | **9.43, 0.0050** |
| RFD-Proportion | 21 | 0.66 (0.4) | 18 | 0.31 (0.4) | **7.37, 0.0100** |
| **Stable vs. Not stable** | **“Stable” at Screening** | | **“Not stable” at Screening** | | |
| Daytime Symptom score | 73 | 0.43 (0.4) | 9 | 0.47 (0.4) | 0.07, 0.7861 |
| Daytime score | 73 | 0.44 (0.4) | 9 | 0.49 (0.4) | 0.14, 0.7133 |
| Nighttime Symptom score | 72 | 0.39 (0.4) | 10 | 0.63 (0.5) | 2.91, 0.0919 |
| Nighttime score | 72 | 0.35 (0.4) | 10 | 0.55 (0.5) | 2.26, 0.1371 |
| Overall Symptom score | 73 | 0.42 (0.4) | 9 | 0.52 (0.4) | 0.47, 0.4946 |
| Number of RFDs | 48 | 3.67 (3.0) | 5 | 1.60 (3.0) | 2.16, 0.1474 |
| RFD-Proportion | 60 | 0.53 (0.4) | 10 | 0.45 (0.4) | 0.25, 0.6219 |
| **Day 1 C-ACT score** | **C-ACT ≥ 20** | | **C-ACT < 20** | |  |
| Daytime Symptom score | 41 | 0.30 (0.4) | 35 | 0.57 (0.4) | **8.10, 0.0057** |
| Daytime score | 41 | 0.33 (0.4) | 35 | 0.57 (0.4) | 6.85, 0.0107 |
| Nighttime Symptom score | 44 | 0.27 (0.4) | 33 | 0.60 (0.5) | **11.82, 0.0010** |
| Nighttime score | 44 | 0.22 (0.3) | 33 | 0.57 (0.4) | **16.74, 0.0001** |
| Overall Symptom score | 41 | 0.30 (0.4) | 35 | 0.58 (0.4) | **10.74, 0.0016** |
| Number of RFDs | 32 | 4.28 (3.1) | 17 | 2.35 (2.6) | 4.66, 0.0360 |
| RFD-Proportion | 37 | 0.63 (0.4) | 28 | 0.41 (0.4) | 4.19, 0.0448 |
| **EOS Day −1 C-ACT score** | **C-ACT ≥ 20** | | **C-ACT < 20** | |  |
| Daytime Symptom score | 40 | 0.29 (0.3) | 30 | 0.68 (0.6) | **12.13, 0.0009** |
| Daytime score | 40 | 0.31 (0.4) | 30 | 0.69 (0.6) | **10.45, 0.0019** |
| Nighttime Symptom score | 39 | 0.24 (0.4) | 28 | 0.52 (0.5) | 6.39, 0.0139 |
| Nighttime score | 39 | 0.21 (0.4) | 28 | 0.48 (0.5) | **7.58, 0.0076** |
| Overall Symptom score | 40 | 0.28 (0.3) | 30 | 0.63 (0.5) | **11.58, 0.0011** |
| Number of RFDs | 31 | 4.39 (3.1) | 20 | 2.30 (2.5) | 6.37, 0.0149 |
| RFD-Proportion | 41 | 0.67 (0.4) | 29 | 0.30 (0.3) | **16.12, 0.0002** |

ANOVA = analysis of variance; C-ACT = Childhood Asthma Control Test; EOS = end of study; ePASD = electronic Pediatric Asthma Symptom Diary; RFD = rescue-medication‒free day; SD = standard deviation.
